# Differential gene expression in abdomens of the malaria vector mosquito, *Anopheles gambiae*, after sugar feeding, blood feeding and *Plasmodium berghei *infection

**DOI:** 10.1186/1471-2164-7-119

**Published:** 2006-05-19

**Authors:** Ali N Dana, Maureen E Hillenmeyer, Neil F Lobo, Marcia K Kern, Patricia A Romans, Frank H Collins

**Affiliations:** 1 Center for Tropical Disease Research and Training, Department of Biological Sciences, University of Notre Dame, Notre Dame, IN 46556, USA; 2 Department of Zoology, University of Toronto, Toronto, ON M5S 3G5, Canada

## Abstract

**Background:**

Large scale sequencing of cDNA libraries can provide profiles of genes expressed in an organism under defined biological and environmental circumstances. We have analyzed sequences of 4541 Expressed Sequence Tags (ESTs) from 3 different cDNA libraries created from abdomens from *Plasmodium *infection-susceptible adult female *Anopheles gambiae*. These libraries were made from sugar fed (S), rat blood fed (RB), and *P. berghei*-infected (IRB) mosquitoes at 30 hours after the blood meal, when most parasites would be transforming ookinetes or very early oocysts.

**Results:**

The S, RB and IRB libraries contained 1727, 1145 and 1669 high quality ESTs, respectively, averaging 455 nucleotides (nt) in length. They assembled into 1975 consensus sequences – 567 contigs and 1408 singletons. Functional annotation was performed to annotate probable molecular functions of the gene products and the biological processes in which they function. Genes represented at high frequency in one or more of the libraries were subjected to digital Northern analysis and results on expression of 5 verified by qRT-PCR.

**Conclusion:**

13% of the 1965 ESTs showing identity to the *A. gambiae *genome sequence represent novel genes. These, together with untranslated regions (UTR) present on many of the ESTs, will inform further genome annotation. We have identified 23 genes encoding products likely to be involved in regulating the cellular oxidative environment and 25 insect immunity genes. We also identified 25 genes as being up or down regulated following blood feeding and/or feeding with *P. berghei *infected blood relative to their expression levels in sugar fed females.

## Background

Sequencing of the *Anopheles gambiae *genome was completed in 2002 [1]. Annotation and gene prediction have been ongoing. Although more than 14,700 genes and more than 16,100 transcripts have now been predicted, the functions of approximately 40% of the gene products remain unknown and *in silico *annotations of many others still require verification [1,2]. Information about the structure, annotation and expression of these genes is necessary for understanding how they are regulated spatially and temporally, and for determining how they function in the mosquito. Large-scale sequencing of cDNA libraries, captures expressed gene products, creating a "molecular snapshot" of the transcriptome. A single sequence read corresponds to the transcript from which it was derived and generates an EST for the underlying gene. Genes can be identified putatively by comparing the derived ESTs with sequences of known annotated genes and gene products.

Large-scale EST sequencing of different cDNA populations provide opportunities for exploration of gene expression under defined biological and environmental conditions. All cells are complex molecular environments regulated by the information in their genes encoding thousands of proteins involved in a multitude of processes. However, only a subset of these genes is actively transcribed at any one time, and in eukaryotes, in any one organ, tissue and cell type. The "digital Northern", an *in silico *form of transcript profiling, can be used to study gene expression by comparing ESTs from clones randomly picked from two or more cDNA libraries created from non-normalized mRNA populations [3-5]. The frequency of any specific sequence should reflect the relative expression level or abundance of that transcript in the libraries [6]. Genes are identified as being differentially expressed using a number of statistical methods [4,7,8]. Finally, the ontology of a gene, the molecular function and biological process in which its product is involved, provide information about the system in which it is expressed.

Infection of the adult female anopheline mosquito with malaria parasites elicits both local and systemic responses from a range of vector organs and tissues. *Plasmodium *infection is also coincident with the ingestion of a blood meal which sets in motion a complex sets of events including digestion and egg production [9-11]. These events involve extensive changes in gene expression in multiple organs, three of which are found in the abdomen, midgut, fat body and ovaries [12-16]. Normal patterns of gene expression in these organs are often significantly further altered in parasitized mosquitoes [1,17,18].

We have investigated genes that are up and down regulated following blood feeding and *Plasmodium berghei *infection of *A. gambiae *females using a direct sequencing approach. Three cDNA libraries were created from the abdomens of sugar-fed, naïve blood-fed, and *P. berghei*-infected females. These whole abdomens contain a multitude of organs, tissues and cell types, and provide an inventory of genes expressed during blood digestion, vitellogenesis and Plasmodium infection. ESTs were obtained and their frequencies compared among the 3 libraries to create transcript profiles. EST annotation using existing databases, BLAST tools and gene ontology classifications yielded information on the most dramatic transcriptional responses of these mosquitoes to blood feeding and parasitism. This catalog of abdominal gene expression will contribute to a more global understanding of anopheline physiology and immunity. It will also provide a resource for improving annotation of the *A. gambiae *genome, thus making it more useful for vector biologists and scientists studying homologous genes in other organisms. Most importantly, increased understanding of anopheline biology at the molecular level may open new avenues for intervention against malaria transmission.

## Results and discussion

Three unidirectionally cloned cDNA libraries were constructed from mRNA isolated from abdomens of *A. gambiae *females that had been fed on 20% sucrose (S library), on rat blood (RB), or on rat blood infected with *P. berghei *(IRB), and then maintained at 19–20°C for 30 h. To determine the staging of *P. berghei *infections in the susceptible 4arr strain, we chose to count melanized transforming ookinetes and early oocysts in the L-35 refractory strain using the rationale that transforming ookinetes/early oocysts are the infection stages being examined in this study whereas counts of later stage oocysts at 5 or 6 d post infection would be likely to underestimate infection intensity at the experimental times points. We acknowledge that this approach assumes that the L35 and 4arr strains experience similar infection time kinetics and similar invasion rates of the midgut by ookinetes, infection attributes that have not been formally established. At 30 h post infection (PI), the majority of parasites in infected L-35 strain mosquitoes were ookinetes still traversing the midgut or transforming to early oocysts on the basal surface of the midgut: the mean number of parasites per *A. gambiae *midgut at 30 h PBM was 10 ± 11 (standard error of the mean, SEM), rising to a peak of 24 ± 23 by 36 h PBM. A *post hoc *Tukey test showed a significant difference in oocyst numbers between these time points, P < 0.05. These values for *A. gambiae *infection by *P. berghei *were similar to infection intensities published elsewhere [19,20]. The unamplified S, RB and IRB libraries contained a total of 3.09 × 10^6^, 1.22 × 10^7^, and 1.13 × 10^6 ^pfu/ml, respectively. The average insert size of 704 clones randomly picked from all three libraries was calculated to be 1068 ± 35 bp (Table 1).

**Table 1:** cDNA LIBRARY INSERT AND EST SIZES*

**Clone Source Library**	**N**	**Mean length (bp)**	**± SE**	**Max. (bp)**	**Min. (bp)**
**S**	243	1003	33	3526	322
**RB**	189	1156	45	3738	152
**IRB**	272	1044	28	2789	317

**Average**		1068	35	3351	264

**EST Source**	**N**	**Mean EST length (nt)^†^**	**± SE**	**Max. (nt)**	**Min. (nt)**

**S singletons**	590	404	8	820	100
**RB singletons**	381	394	8	748	100
**IRB singletons**	437	377	8	785	100
**Contigs**	567	595	11	1585	104

**Average**		455	9	1039	101

*Library abbreviations are defined in the text. N = number of clone inserts, ESTs sequenced ^†^The mean EST length in nucleotides (nt) is the length following end-trimming, short sequence filtering and EST contig assembly.

SE = standard error of the mean.

**Figure 1:**
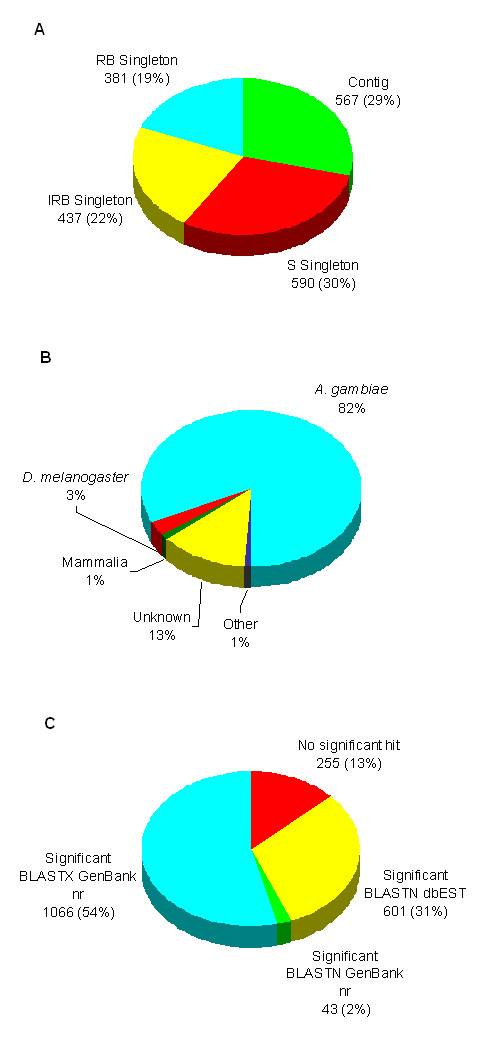
Distributions of ESTs among the S, RB and IRB libraries. A total of 1408 singletons, unique transcripts, and 567 multi-EST contigs was examined. A. Distribution of singletons among libraries relative to total contigs. The % of 1975 singletons and contigs is shown in brackets following the number of singletons within each library. B. Taxonomic distribution of all 1975 assembled sequence homologies following BLASTX and BLASTN searches of GenBank nr and a BLASTN search of dbEST. C. Identification of 1965 transcripts sharing *A. gambiae *genomic identity, E < 10^-4^.

cDNA inserts from 3264, 1920, and 3456 white plaques randomly picked from the S, RB, and IRB libraries, respectively, were amplified by PCR and sequenced from their 5' ends. The resulting ESTs were filtered based on sequence trace file quality, screened for mitochondrial contamination and assembled into contigs using SeqMan II. Following the initial SeqMan assembly, only high quality non-mitochondrial sequences >100 bp, corresponding to 1736, 1216, and 1772 ESTs from each of the S, RB and IRB libraries, respectively, were analyzed further. Their average length was 455 ± 8 nt (Table 1). These 4724 high quality ESTs assembled into a total of 1989 contigs and singletons. Sequence identity searches showed that 4 contigs and 10 singletons were of non-mosquito origin: these sequences shared no identity with the *A. gambiae *genome [21] but did with proteins such as rat alpha and beta hemoglobin chains. When these contaminants had been removed, a total of 4541 high quality ESTs remained for further analysis, 1727 from S, 1145 from RB, and 1669 from IRB. They assembled into 1975 consensus sequences, 567 contigs and 1408 singletons (Table 1, Figure 1A). Each was given a unique assembled sequence (AS) number. These EST sequences can be accessed through the NCBI EST database, dbEST [22].

The EST assembly may have estimated the number of unique genes in the libraries inaccurately due to sequencing errors, sequence polymorphisms, alternate splicing of transcripts, and lack of overlap of 3'and 5' sequences representing the same gene product [22,23]. To evaluate this inaccuracy, we compared sequences sharing the same top BLAST hit by aligning them with the nucleotide sequences of their predicted genes using CLUSTAL W [24,25] and its default parameters. Of the 974 assembled sequences that shared identity with predicted *A. gambiae *proteins, 65 aligned with a protein already represented in the data set. When assembled sequences identified as being from the same gene failed to align, they failed to do so mainly because their sequences did not overlap, i.e., they were from different parts of a gene. Alternative splicing of transcripts and sequence dissimilarity in excess of contig assembly thresholds also contributed to these alignment failures. We estimate that the total number of assembled sequences may have overestimated the total number of unique genes by approximately 4%.

### Sequence identity searches

The 1975 assembled *A. gambiae *EST contig and singleton sequences were distributed among the three libraries as shown in Figure 1A. They were searched against nucleotide and protein databases for identity to known genes and proteins using BLAST algorithms. 82% of them showed highest identity to predicted or identified *A. gambiae *genes, 3% to *Drosophila melanogaster *genes, 1% to mammalian genes, 1% to other taxonomic groups, while 13% remained unknown after this analysis (Figure 1B) BLASTN alignments identified 1965 consensus sequences with significant identity (<E^-4^) to the *A. gambiae *genome assembly and 10 that did not (Figure 1C). The 1965 consensus sequences identified as being derived from *A. gambiae *are composed of 1710 sequences which shared identity with publicly available nucleotide and/or amino acid sequences from other organisms and 255 sequences which showed no identity to any sequence in the GenBank nr and dbEST databases. 95% (1620/1710) of the *A. gambiae*-derived consensus sequences exhibited identity with *A. gambiae *protein sequences predicted *in silico *from the genome sequence and sequences submitted to databases prior to genome sequencing (data not shown). These included 974 sequences with identity to predicted proteins, 48 with identity to protein sequences derived from other submitted gene sequences, and 598 with identity to ESTs. It is surprising that no Plasmodium ESTs were identified.

Only 49.6% (974/1965) of assembled sequences sharing identity with the *A. gambiae *genome also shared identity with predicted *A. gambiae *proteins. The remaining 991 sequences may be derived from 5' or 3' untranslated regions (UTRs), may lack an ORF due to frame shift errors occurring during cloning or sequencing, may be 5' truncated, or may be just too short to be identified, despite the 100 nt cutoff. They may also represent novel genes. Holt *et al*. [1] concluded that more than 1300 genes might have escaped prediction in the first annotation of the *A. gambiae *genome. The current gene number prediction is 14,707 [2], an increase of more than 1000 from the original estimate. Computational techniques may inaccurately predict genes by missing exons derived solely from promoters, or that are due to alternative splicing of transcripts or to use of non-canonical splice sites, alternative translational initiation and/or polyadenylation sites [26]. Thus, the majority of ESTs generated in this study may enhance gene prediction in the *A. gambiae *genome through refinement of existing gene models and providing evidence for new ones.

By definition, ESTs are generally short sequences of approximately 300–500 nt derived from transcripts [27]. The sequences assembled in this study had an average length of 455 nt and many could have consisted mainly of 5'- or 3'-UTRs. The average lengths of the 5'- and 3'-UTRs in Release 3 of the *Drosophila *genome were 265 and 442 nt respectively, and the average ratio of their length/coding sequence was 0.75 [28]. Accordingly, BLASTN sequence identity searches were performed against dbEST to annotate assembled sequences that might have lacked a predicted ORF because they consisted mainly of either a long 5'-UTR, or of 3'-UTR resulting from 5' truncation of transcripts during cDNA library construction. dbEST contains more than 100,000 *A. gambiae *ESTs, the majority of which were generated from large-scale sequencing of two non-normalized cDNA libraries constructed from non blood-fed and blood-fed whole adult females [1]. 601, or 66.9% of the 898 unique gene products sharing identity with the genome but not identified by BLASTX search of GenBank Nr were identified by this BLASTN search of dbEST. 99.5% of these shared sequence identity with at least one *A. gambiae *EST. After completion of all identity searches, 255 assembled sequences still failed to show significant identity with the ESTs in dbEST or with the predicted proteins in GenBank Nr, though they did with the *A. gambiae *genome. These appear to be truly novel.

Since *ab initio *gene prediction programs used to analyze genomes can only identify open reading frames (ORFs), cDNA sequences provide an essential tool for properly validating gene identification and annotation. Misra *et al*. [28] reported that reannotation of the *Drosophila *genome following Release 3 resulted in much-improved prediction of alternatively spliced transcripts and annotation of UTRs due to the increased number of ESTs and cDNAs available. The reannotation resulted in changes to 85% of gene models, including major changes in 40% of predicted proteins, without significantly affecting the number of genes predicted. Since the untranslated regions of transcripts contain sequences influencing transcript fates including subcellular localization and mRNA turnover, as well as *cis*-regulatory information, a database of nucleotide sequences corresponding to predicted transcripts that includes UTRs may provide a better tool for EST and genome annotation than a database of predicted proteins. Most importantly, however, accurate identification of UTRs and alternative patterns of intron splicing in the *A. gambiae *genomic sequence that can be obtained through EST projects such as this one are necessary for ultimately understanding gene regulation at the post-transcriptional level.

### Functional annotation of ESTs

The adult female mosquito abdomen contains several complex organs and tissues including the midgut, the ovaries and the fat body. These function in the normal processes of blood meal digestion and egg production, as well as in responses to infection by and defense against pathogens. Functional annotation of the abdominal genes represented in the three cDNA libraries was performed to gain insight into the physiological events required for reproduction and the pathological ones induced by infection with *Plasmodium*. Molecular function and biological process were assigned to the consensus sequences based on sequence similarity to known genes and proteins and to the existing gene indices for *A. gambiae *and *D. melanogaster*, TIGR Gene Index and GadFly, respectively.

The 1975 gene products predicted after EST clustering were categorized into 8 major biological processes with 34 subdivisions. A 9^th ^category, Unknown, represents gene products with no ascribable function. The library-specific results of these functional assignments are in Table 2. More detailed annotation of all 1975 gene products are provided [see Additional file 1]. For all three libraries taken together, the largest category, representing 1329 gene products or 67.3% of the total, remains the "Unknown". The three next most numerous categories are "Metabolism", 441 unique transcripts, 23.3% of the total; "Protein Synthesis", 418, 21.2%; and "Egg Production", 81, 4.1%. The largest represented subdivision is "Translation". Perhaps unexpectedly, the genes likely to be involved in egg production are represented in similar proportions among the three libraries. While it is possible that this result is an artifact created by timing the mRNA samples used for library creation to 30 hr PBM at 19°C, a time close to the peak of vitellogenic activity at this temperature, the gene products involved in oogenesis may also play other roles in the life cycle of the mosquito. For instance, some of the gene products in the Toll pathway, a signaling cascade that controls dorsal-ventral patterning of the *Drosophila *embryo during development [29], are also important in the induction of several immune-related peptides [30-33].

**Table 2:** BIOLOGICAL PROCESSES OF GENES REPRESENTED IN LIBRARIES*

	Total S	Total RB	Total IRB
Metabolism			
Simple/Complex Carbohydrate Metabolism and Transport	21 (28)†	15 (17)	13 (15)
Oxidative Phosphorylation	41 (64)	38 (61)	30 (69)
Lysosomal Enzymatic Digestion	1 (2)	0	4 (4)
Protein Digestion	13 (63)	7 (49)	11 (63)
Protein Modification, Metabolism, Transport and Localization	42 (54)	38 (49)	39 (63)
Amino Acid and Derivative Metabolism and Transport	10 (13)	6 (10)	16 (20)
Nucleobase/Nucleoside/Nucleotide/Nucleic acid Metabolism and Transport	9 (21)	10 (12)	13 (19)
Fatty Acid/Lipid Metabolism and Transport	9 (11)	8 (8)	5 (6)
Vitamin/Vitamin Derivative/Cofactor Metabolism and Transport	2 (3)	2 (3)	2 (2)
Xenobiotic Metabolism and Transport	5 (5)	7 (8)	5 (6)
Pigment Synthesis and Transport	1 (1)	2 (2)	4 (5)
Other	3 (4)	6 (6)	3 (3)

Total	157 (269)	139 (225)	145 (275)

Transport			
Ion Transport	10 (11)	14 (16)	12 (14)
Receptor-mediated Endocytosis	7 (10)	4 (4)	6 (11)

Total	17 (21)	18 (20)	18 (25)

Protein Synthesis			
Transcription and mRNA Processing	17 (23)	19 (20)	19 (21)
Translation	108 (436)	97 (325)	115 (482)
Protein Folding	14 (24)	19 (21)	10 (13)

Total	139 (483)	135 (366)	144 (516)

Cellular Processes			
Cell Cycle	14 (17)	10 (13)	6 (11)
Cellular Proliferation	4 (4)	2 (2)	3 (3)
Chromatin Assembly/Disassembly	4 (6)	5 (7)	5 (10)
Apoptosis	2 (2)	2 (2)	5 (7)

Senescence	2 (3)	0	1 (2)

Viral Life Cycle	1 (1)	1 (1)	0

Total	27 (33)	20(25)	20 (33)

Egg Production			
Vitellogenesis/Oogenesis/Embryogenesis	29 (33)	24 (40)	27 (60)
Melanization	0	1 (1)	0

Total	29 (33)	25 (41)	27 (60)

Cellular Communication			
Signal Transduction	11 (14)	5 (7)	6 (12)
Cell-cell Signaling	4 (7)	4 (4)	2 (3)

Total	15 (21)	9 (11)	8 (15)

Intra-/Extra-cellular Architecture Maintenance			
Structural	8 (35)	11 (24)	6 (30)
Muscle-related	6 (6)	4 (5)	7 (8)
Cell Adhesion	3 (3)	3 (3)	1 (1)
Cytoskeleton Organization and Biogenesis	4 (6)	12 (17)	12 (18)

Total	21 (50)	30 (49)	26 (57)

Response to Stress/External Stimulus			
Response to Oxidative Stress	7 (9)	8 (11)	14 (29)
Immune/Defense Response	11 (29)	9 (15)	14 (20)
Chemosensory Perception	3 (3)	1 (1)	1 (1)

Total	21 (41)	18 (27)	29 (50)

Unknown			
Total	573 (776)	326 (415)	430 (663)

*This analysis does not take into account that some genes are represented in more than 1 library or that more than one biological process may have been ascribed to particular genes.

†Number of Contigs containing one or more ESTs (total ESTs/genes).

The first large scale studies to identify genes involved in mosquito responses to *Plasmodium *infection relied on cDNA libraries prepared from bacteria-challenged mosquito tissues [34,35]. Three more recent studies have reported direct screens for *Anopheles *genes responding to *Plasmodium *infection [17,36,37]. Our IRB library has provided an additional opportunity to study transcripts whose abundance may be regulated by infection with *P. berghei*. It showed an increase in the proportion of gene products present in biological processes likely to be responses to parasite infection, including responses to oxidative stress and immunity-related defenses. Some transcript profiles looked at here are based on the comparison of ESTs that vary by only one unit. Though this may be considered to be of limiting value, it is important to note that most profiles are consistent with previous studies and are discussed individually. In addition, the tissues used here are composed of whole abdomens, which contain gut, blood cells, fat body, epidermis, ovaries and other cell and tissue types. Differences of gene expression patterns between this and previous studies may relate to the somewhat different tissues that were assayed.

Reactive oxygen species may be generated through the activities of nitric oxide synthase (NOS) and peroxidases [38,39]. Transcripts encoding number of enzymes involved in regulating the cellular oxidative environment were identified in all three libraries. These include multiple glutathione S-transferases (GSTs), peroxidases, and peroxiredoxin (Table 3), but not nitric oxide synthase (NOS). Failure to identify inducible NOS transcripts at least in the IRB library may relate to the abundance and or timing of its transcription though *P. berghei *invasion induced NOS both systemically and locally in the midgut in *Anopheles stephensi *24–48 hours post infection (PI) [40]. In parasite-damaged midgut cells, the increase in NOS levels was concurrent with other morphological changes associated with apoptosis [41]. NOS is known to be activated transcriptionally in *A. gambiae *within 22–24 hr PI with *P. berghei *[42,43]. Activation is mainly in the midgut, as expected for this time period.

**Table 3:** OXIDATION AND STRESS RESPONSE GENES

**AS**	**# S ESTs**	**# RB ESTs**	**# IRB ESTs**	**Total # ESTs**	**Genome Scaffold**	**Start**	**End**	**E value**	**Blast Type**	**Accession**	**Blast Hit**	**E value**	**Putative Identity**
34	2	1	5	8	8898	1665469	1665984	0	X	EAA09273	agCP14153	1.00E-111	*glutathione S-transferase D11*
35	0	4	3	7	8880	1407780	1408378	0	X	Q93113	glutathione S-transferase 1–6	1.00E-110	*glutathione S-transferase 1–6*
172	2	0	2	4	8816	4859270	4859540	1E-130	X	EAA05108	agCP6896	7E-65	*Glutaredoxin (GRX1)*
307	0	1	0	1	8986	5275996	5276383	1E-173	X	EAA00516	agCP9336	8E-46	*copper ion transporter*
620	0	0	2	2	8984	9812533	9812822	1E-161	X	AAL58538	^*glutathioneS-*^transferase E3	1E-73	*glutathione S-transferase E3*
622	0	0	1	1	8980	538441	538745	1E-172	X	AAF68382	thioredoxin 1	5E-49	*thioredoxin 1 glutathione S-*
704	0	0	1	1	8880	1406678	1407026	0	X	EAA08605	agCP2490	4E-36	*transferase 1–6*
909	0	1	0	1	8984	9810450	9810635	1E-101	X	AAG45163	^*glutathioneS-*^transferase E1	2E-20	*glutathione S-transferase 3–1*
910	0	0	1	1	8984	9809004	9809209	1E-108	X	AAG45164	^*glutathioneS-*^transferase E2	6E-28	*glutathione S-transferase E2*
931	0	1	0	1	8933	621123	621383	1E-145	X	EAA09899	agCP11759	1E-73	*Manganese Superoxide dismutase 1*
1012	0	0	4	4	8849	2536282	2536794	0	X	EAA07169	agCP10692	6E-80	*Superoxide dismutase 3-D (Cu,Zn)*
1041	1	0	2	3	8849	1674668	1675054	0	X	EAA07207	agCP10713	1E-115	*glutathione S-transferase S1-2*
1042	0	1	2	3	8986	8102109	8102313	1E-105	X	EAA00332	agCP9864	2E-90	*1-cys peroxiredoxin TPX4*
1065	0	0	3	3	8804	83740	83869	6E-45	N	AJ284424	4A3B-AAW-E-09-F A. gambiae immune competent 4A3B	2E-51	*2-Cys thioredoxin peroxidase TPX2*
1482	0	0	1	1	8984	9812211	9812365	2E-74	X	AAL58538	^*glutathioneS-*^transferase E3	8E-06	*glutathione S-transferase E3 2-Cys*
1684	0	1	0	1	8804	84431	84661	1E-117	X	EAA03855	agCP1990	2E-35	*thioredoxin peroxidase TPX2 thioredoxin 1*
1909	1	0	0	1	8980	539114	539324	1E-109	N	AJ283949	4A3B-AAH-C-12-F A. gambiae immune competent 4A3B	1E-107	
2033	1	0	0	1	8898	1672566	1673171	0	X	EAA09147	agCP14131	1E-120	*glutathione S-transferase D3*
2078	1	0	0	1	8984	9807310	9807705	0	X	AAL59653	^*glutathioneS-*^transferase E4	4E-71	*glutathione S-transferase E4*
2127	0	1	0	1	8986	8102593	8102854	1E-13i6	X	EAA00332	agCP9864	6E-31	*peroxiredoxin TPX4*
2156	0	0	1	1	8880	3392447	3392665	1E-120	X	EAA08586	agCP2356	2E-43	*thioredoxin*
2296	0	0	1	1	8807	1924679	1924904	1E-110	X	EAA03983	agCP3166	5E-75	*thioredoxin peroxidase TPX3*
2334	1	0	0	1	8880	3614672	3614912	1E-124	X	EAA08535	agCP2389	3E-58	*glutathione peroxidase 2-A, 2-B*

Although GSTs are most often associated with the detoxification of xenobiotics they are also involved in a number of cellular processes including protection from oxidative stress and apoptosis [44]. In addition to regulating thioredoxin, GSTs regulate the redox state of pro-apoptotic proteins [29,45] These redox-modulating enzymes may be inducing oxidative stress either in response to *Plasmodium*-induced inflammation or to apoptosis of mosquito tissues. Thus, the generation and modulation of reactive oxygen species by multiple enzymes induced later in *A. gambiae *infection, which we now observe, may regulate or limit oocyst development.

Transcripts of 25 genes involved in insect immune responses were identified (Table 4). Previously characterized *A. gambiae *immune-related peptides included AS 1286, gram negative [bacteria] binding protein, GNBP; AS 2178, cecropinA, CecA; and AS 1197, cecropinB, CecB. Two ficolins, AS 1364 and AS 1922, and four lysozymes AS 32, AS 221, AS 659 and AS 2030, were also identified. GNBP, has been linked with the immune response to *Plasmodium *infection, had already been found to be induced in the 20–30 h following *A. gambiae *infection with *P. berghei *[35,46]

**Table 4:** IMMUNE-RELATED/DEFENSE RESPONSE GENES

**AS**	**# S ESTs**	**# RB ESTs**	**# IRB ESTs**	**Total # ESTs**	**Genome Scaffold**	**Start**	**End**	**E value**	**Blast Type**	**Accession**	**Blast Hit**	**E value**	**Putative Identity**
28	17	3	0	20	8987	14943052	14943642	0	X	EAA01687	agCP11956	1E-142	serine protease
32	2	3	4	9	8807	3383097	3383307	1E-116	X	Q17005	Lysozyme precursor (1,4-beta-N-acetylmuramidase)	5E-67	lysozyme precursor (1,4-beta-N-acetylmuramidase)
68	1	0	0	1	8794	217313	217497	1E-100	X	EAA02509	agCP11665	3E-13	serine protease 14D2
126	1	0	0	1	8964	2322618	2322792	2E-86	X	EAA12171	agCP10937	1E-25	TEP3
153	2	0	0	2	8986	8953596	8953709	4E-45	N	BM621296	17000687446469 A.Gam.ad.cDNA1	2E-63	fat-spondin
180	1	0	1	2	8859	3227518	3227913	0	X	EAA07758	agCP1111	4E-70	signal transducer
221	0	0	1	1	8807	3388356	3388647	1E-158	X	EAA04406	agCP3675	8E-79	lysozyme c-8
418	1	0	3	4	8944	1827235	1827637	0	X	EAA10153	agCP15402	3E-64	gambicin
659	0	0	1	1	8807	3140140	3140290	5E-80	X	EAA04458	agCP3967	3E-56	lysozyme c-4
804	0	1	0	1	8960	17845546	17845795	1E-139	X	CAA09389	ICHIT protein	5E-31	ICHIT
832	1	0	1	2	8811	1718220	1718532	1E-151	N	BM635649	17000687559053 A.Gam.ad.cDNA1	3E-64	AgToll
995	0	2	2	4	8960	23616	23997	0	X	EAA11001	agCP5701	1E-30	serine protease
1049	0	2	0	2	8944	1995046	1995300	1E-138	X	EAA10138	agCP15205	1E-78	TEP12
1095	1	1	0	2	8960	16595875	16596267	0	X	EAA11334	agCP6381	7E-30	signal transducer
1120	0	1	1	2	8986	8676449	8676732	1E-159	X	EAA00414	agCP9557	5E-31	serine protease
1197	0	0	1	1	8847	1123621	1123927	1E-173	X	EAA06859	agCP7366	3E-25	Cecropin B
1286	0	0	1	1	8898	2844394	2844678	1E-149	X	EAA09116	agCP14093	4E-28	GNBPB1
1364	0	0	1	1	8948	918161	918491	1E-166	X	EAA10406	agCP2049	2E-54	ficolin
1616	0	1	0	1	8980	7957050	7957284	1E-128	X	CAB90818	serine protease	7E-79	serine protease
1701	0	1	0	1	8794	212210	212531	0	X	AAB62929	serine protease 14D	2E-56	serine protease 14D
1922	0	0	1	1	8816	1069890	1070396	0	X	EAA05160	agCP6864	2E-55	ficolin
1997	1	0	0	1	8859	9665976	9666551	0	N	AF444782	AgToll9	4E-06	AgToll9
2030	0	0	1	1	8807	3137120	3137454	0	X	EAA04667	agCP3164	3E-82	Lysozyme c-7
2038	1	0	0	1	8975	72415	72691	1E-155	N	AJ420785	spi21F gene	1E-71	serpin spi21F
2178	0	0	1	1	8847	1121909	1122047	6E-73	X	EAA06858	agCP7503	6E-21	Cecropin A

Cecropins are small, basic peptides which cause lysis of gram negative and some gram positive bacteria by forming pores in their inner cell membranes (see [47] for review). They have been found in a wide variety of insects, including many vectors of parasitic diseases. The *A. gambiae *genome contains either 3 or 4 cecropin genes [48,49]. The *CecA *gene product, Cecropin A, is induced by *Plasmodium *during the early stages of infection [48,50]. The divergently transcribed *CecA *and *CecB *genes are both up-regulated in an *A. gambiae *cell line after challenge with lipopolysaccharide and heat-inactivated bacteria [49]. This induction is regulated by a currently uncharacterized NF B-class transcription factor. Since these two *Cec *genes were identified only in the IRB library, they may be involved specifically in anti-*Plasmodium *responses.

Ficolins are carbohydrate-binding proteins related to collectins, a class of innate immunity lectins involved in the phagocytic ingestion of apoptotic cells [51-53]. In vertebrate innate immune responses, ficolins initiate the lectin pathway of complement activation [54]. The two ficolins we identified showed highest amino acid identity to the *Drosophila *Ficolin 2 precursor, but they correspond to different *A. gambiae *gene products located on different chromosomes *in silico*. Since they were found only in the IRB library, it is likely that they are involved in *Plasmodium *recognition prior to immune activation unless recognition, which appears to involve multiple pattern recognition proteins, is progressive.

The lysosome contributes to cellular maintenance through involvement in autophagy and to immunity through protease-mediated degradation of phagocytosed substances and apoptosis-like programmed cell death [55]. Since all lysosomal enzymes identified in this study except for AS 32 were found solely in the IRB library, the increase in lysosomal proteases in the IRB library may be indicative of phagocytic, inflammatory, and/or apoptotic responses to *Plasmodium *infection. We identified transcripts of four different lysozyme genes among our ESTs. AS 32, for which we had 9 ESTs distributed in all three libraries, showed clear evidence of alternative splicing. This gene corresponds to the previously characterized basic lysozyme gene, ENSANGG00000019898 [56]. They had concluded that this gene was expressed much more abundantly in sugar-fed than in blood-fed *A. gambiae*. However, the primers they used for their RT-PCR would also have amplified transcripts from a more recently identified lysozyme gene, ENSANGG00000015399, agCP3675, (AD, unpublished), thus potentially confounding their results. AS 221, AS 659 and AS 2030, the three other lysozyme EST sequences corresponding to ENSANGG00000015399 (agCP3675), ENSANGG00000015906 (agCP3967), and ENSANGG00000015950 (agCP3164) were all found at one EST each in the IRB library. Thus, among abdominal cDNAs expressed following an IRB, we have identified transcripts representing 4 of the 5 lysozyme genes and 5 of the 7 potential lysozyme transcripts encoded in the *A. gambiae *genome. It is interesting that all of the *A. gambiae *lysozyme genes are located close together in the same region of chromosome 2L and that their promoters all contain potential binding sites for NFB -like transcription factors, as would be expected for pathogen-induced transcription. Since AS 221 has also been identified as being induced more than two-fold at 48 h after an uninfected blood meal in our microarray study [12], it is also possible that different lysozymes or combinations of lysozymes may act as antibacterial agents in *A. gambiae *following sugar and blood feeding.

Lysozymes were not identified as potential immunity-related proteins by Christophides *et al*. [48]. However, Hultmark [57] suggested that lysozyme may well be an immune protein that acts synergistically with cecropin to release microbial surface components, since its activity increases in concert with cecropin activity in bacteria-challenged silk worm larvae [58]. Therefore it is possible that multiple *A. gambiae *lysozymes may act together with cecropin(s) in an anti-*Plasmodium *response. The overall complexity of lysozyme genes, transcripts, and potential induction patterns in *A. gambiae *suggest that their roles as immune mediators deserve additional study in this mosquito.

AS 418, Gambicin, transcripts were found in both the S and IRB libraries, insignificantly more in the latter (Table 4). The gambicin gene encodes a 6.8 kDa antimicrobial peptide unique to *A. gambiae *[59]. Gambicin transcripts were found primarily, but not exclusively in the anterior midguts of both sugar-fed and blood-fed adult female mosquitoes. RT-PCR suggested that its transcription is induced slightly above basal levels at 30 h PBM and by about 3-fold by 24 h post infection, at which point *P. berghei *ookinetes are invading the midgut epithelium.

The association of AS804, ICHIT, a galectin with chitin-like domains, only with the RB library was unexpected. This is because ICHIT transcripts were found to be abundant in the midguts of sugar-fed adult female mosquitoes and only weakly induced in *A. gambiae *midguts 24 h PI with *P. berghei *[42].

Two Toll receptor gene transcripts were identified (Table 4). The Toll signal transduction pathway is involved both in insect immune responses, and, in *Drosophila*, at least, in specification of the dorsal-ventral body axis during embryogenesis. While there are 11 known Toll receptor genes in *A. gambiae *[48], the expression of only 4 has been characterized [60]. One, AS 832, corresponding to *AgToll*, was found only in the S and IRB libraries. Luna *et al*. [60] demonstrated that this gene is abundantly expressed in ovaries, and not at all in midgut. They also found that this gene is only weakly induced by bacterial challenge. This gene is actually duplicated in the *A. gambiae *genome as *AgToll1 *and *AgToll1B*, both of which are closely related to *AgToll5A *and *AgToll5B*, as well as to *D. melanogaster Toll, DmToll1*, encoding the receptor mediating body axis formation, and to *DmToll5 *[32].

Consequently, despite the fact that AS 832 was not found in the RB library, its primary function is more likely to be in embryogenesis than in defense. AS 1997, *AgToll 9*, was found only in the S library. It is abundantly and specifically expressed in midgut during multiple developmental stages [60], and is weakly induced in larvae by bacterial challenge, though it has not been tested for induction in *Plasmodium*-infected mosquitoes. It and its *Drosophila *ortholog, *DmToll9*, are most closely related to mammalian TLR genes, and may well be involved in immunity functions.

Serine proteases (SPs) and serine protease inhibitors (serpins, SRPNs) the inhibitors of SPs, function in multiple processes, of which immunity is only one. AS 68, Sp 14D2; AS 28, SP G13; AS 153, fat-spondin; AS 2038, a serpin transcribed from the spi21F/SRPN10 locus, were all unique to the S library. The majority, 20/31, of SP transcripts identified, 17 of them derived from AS 28, SP G13, were from the S library (Table 4). Sp14D2 is abundantly expressed in adult females at 4 days post eclosion and is induced only slightly following bacterial challenge and *P. berghei *infection [61,62]. It may not be involved in immune responses. The adult gut-specific non-trypsin SP G13 is found in both blood fed and non-blood fed females, and is also immune responsive in bacterially challenged larvae [34]. The SRPN10 gene encodes four alternatively spliced transcripts which are differentially expressed in the midgut during development and following microbrial challenge [63]. We were unable to determine which one of these transcripts corresponds to AS 2038.

Four SPs were found in the RB library in addition to the previously described SP G13 (Table 4). These are AS 170, Sp14D1; AS 1616, CLIPB15; AS 1120, CLIPA7; and AS 995, agCP5701, previously predicted only computationally. Sp14D1 transcripts are expressed constitutively in multiple mosquito stages [62]. In adult females, they are expressed in the ovary and fat body, but not in the midgut. The Sp14D1 gene is induced by 24 h PBM and after a bacterial challenge. Therefore this serine protease may have roles both in development and in immunity. CLIPB15 showed significant induction following bacterial challenge and during malaria parasite invasion [48]. CLIPA7 and agCP5701 were found in both the RB and IRB libraries. CLIPA7 has not yet been characterized. However, the agCP5701 sequence shares identity with Ssp3, a serine protease recently identified in the hematophagous fly, *Stomoxys calcitrans *[64]. This serine protease colocalizes with defensin and is thought to activate it.

It is well known that insect immunity-effector genes are not necessarily induced by pathogen challenge, but instead, may be constitutively expressed in situations in which pathogens could be encountered. Certainly, defensin and lysozyme are constitutively expressed during blood feeding in ticks [65-68]. Thus several of the SP genes represented in our libraries may have immune functions, even though none is unambiguously upregulated following *P. berghei *infection.

Fat-spondin is a serine protease inhibitor of the Kunitz family that is down-regulated following septic injury. It is also regulated by Spaetzle, the activator of the Toll pathway in *Drosophila *[30,31]. Therefore mosquito fat-spondin may regulate immune responses during nectar feeding.

### Digital Northern and verification of selected gene expression patterns by qRT-PCR

The complement of genes expressed in a cell or tissue represents its transcriptome [69]. Transcriptome analysis has been approached in several ways. Okubo *et al*. [5] demonstrated that a non-normalized, non-amplified cDNA library can faithfully represent the mRNA population in a tissue, and that such a resource could be used to explore the diverse array of active genes and their mRNA abundances in a tissue. Thus, the "digital Northern" became one of the first genome scale analytical methods employed in the study of gene expression. Three classes of mRNA transcript abundance were recognized based on reassociation kinetics; high abundance (5–15 mRNA species at ~10,000 copies per cell), intermediate abundance (500 species at ~300 copies per cell) and low abundance (10,000 different species at 1–15 copies per cell) [70-72]. Lee *et al*. [73] showed that in a random sample of approximately 3000 ESTs from a single cDNA library, > 99% of highly abundant transcripts, 85% of intermediate abundance transcripts, and < 5% of low abundance transcripts should be represented at least once. Hwang *et al*. [4] demonstrated that differentially expressed genes could be identified digitally even in small data sets although the analysis would necessarily be restricted to more abundantly expressed transcripts, those that are expressed at a frequency of greater than 1 transcript in 800.

Since our three cDNA libraries were neither normalized nor amplified, the number of ESTs in a contig should represent the abundance of the corresponding transcript in the libraries. Therefore we used the digital Northern technique to identify genes differentially expressed in the abdomens of *A. gambiae *females in response to a sugar meal, to a blood meal and to a blood meal containing infective malaria parasites. Although our investigation is necessarily limited to the analysis of moderately and highly abundant mRNAs, it offers a unique opportunity to identify a diverse array of transcripts and to examine some of the greater fluctuations in transcript abundance between and among libraries.

Although large EST sequencing experiments are not repeated and consequently do not exhibit variation, the number of ESTs corresponding to particular transcribed genes approximates a Poisson distribution [74]. Consequently two statistics, the Audic and Claverie Statistic and the R statistic, both based on a Poisson distribution, are used to evaluate the results of these experiments. The Audic and Claverie statistic is based on the assumptions that identifying any specific cDNA in a library is a rare event that represents one possible outcome of many, and that the total number of possible outcomes is unknown [7]. Confidence intervals, both 1% and 5%, corresponding to the likelihood of selecting a specific species of cDNA among a subset of all possible cDNAs, are generated. The probability of selecting a specific clone is independent of sample size because the statistic accounts for differences in population size between libraries. This statistic becomes more reliable as the size of the sample analyzed increases. However, it is applicable only to pairwise comparisons and cannot be used to identify transcripts differentially expressed in more than two libraries. Stekel *et al*. [8] proposed using the R statistic to analyze the abundances of cDNAs in multiple libraries. The R statistic is a log likelihood ratio and similarly to the C^2 ^distribution, its distribution is asymptotic. This log likelihood ratio is constructed from the likelihood of seeing an observed event, or in this case, the probability of selecting a specific species of cDNA from multiple libraries. It represents the differences in observed EST counts among multiple libraries as differences in gene expression levels rather than as random sampling variability.

**Figure 2:**
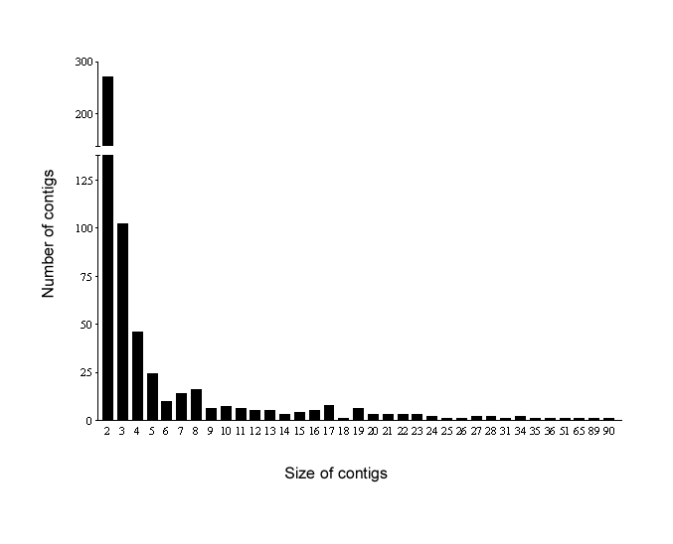
Transcript abundance distribution within the 567 multi-EST contigs of the S, RB and IRB libraries. The 1408 singletons are not shown. Contig size = the number of ESTs contained within it.

Consequently we made pairwise comparisons in gene expression frequency using the Audic and Claverie Statistic and identified genes as differentially expressed among the three libraries using the R statistic. Contig sizes varied significantly (Figure 2). Almost half of the contigs contained only 2 ESTs, although the maximum number was 90. As expected, the number of contigs containing larger numbers of ESTs decreased exponentially in frequency as the number of contained ESTs increased.

Housekeeping genes are usually constitutively expressed in virtually all cell types of a multicellular organism even under a wide range of physiological and experimental circumstances [5]. Typically, though there are exceptions, they are also abundantly expressed. Thus, contigs represented in all three libraries and containing higher numbers of ESTs are most likely to represent transcripts encoding proteins involved in housekeeping functions. Contigs composed of more than 20 ESTs are described in Table 5. Indeed, 18/29 ASs, approximately 62% of contigs containing more than 20 ESTs, encode structural constituents of ribosomes. Unexpectedly, other genes commonly considered to housekeeping genes, such as those involved in oxidative phosphorylation, are not represented among these contigs. In fact, the most frequently identified gene products were AS 3, the *A. gambiae *agCP10095, and AS 1, a homolog of *D. melanogaster *LP07070, with 89 and 90 ESTs respectively. Surprisingly for abundant transcripts likely to encode housekeeping functions, there are currently no clues in the literature as to their functions. One potential clue is that transcription of AS 1 and AS 3 may be induced by *P. berghei *infection. It is also noteworthy that three of the contigs containing more than 20 ESTs, ASs 28, 99, and 18, represent genes encoding serine-type endopeptidases. These enzymes all share high sequence identity with previously studied serine proteases and are differentially expressed among the three libraries (see below).

**Table 5:** MOLECULAR FUNCTIONS OF CONTIGS CONTAINING > 20 ESTS.

**Contig**	**S ESTs**	**RB ESTs**	**IRB ESTs**	**Total ESTs**	**Blast Hit**	**Molecular Function**	**Organism**
7	3	8	9	20	agCP11398	structural constituent of ribosome	*A. gambiae*
14	11	6	3	20	agCP1538	structural constituent of ribosome	*A. gambiae*
**28**	**17**	**3**	**0**	**20**	**agCP11956**	**serine-type peptidase**	***A. gambiae***
140	5	6	10	21	agCP7468	structural constituent of ribosome	*A. gambiae*
167	11	4	6	21	RpS27A	structural constituent of ribosome	*D. melanogaster*
**516**	**8**	**10**	**3**	**21**	**agCP3409**	**peritrophin**	***A. gambiae***
**5**	**9**	**4**	**9**	**22**	**agCP12023**	**unknown**	***A. gambiae***
20	13	5	4	22	agCP10687	structural constituent of ribosome	*A. gambiae*
**201**	**7**	**6**	**9**	**22**	**agCP7935**	**unknown**	***A. gambiae***
8	15	5	3	23	agCP8133	structural constituent of ribosome	*A. gambiae*
**13**	**16**	**2**	**7**	**23**	**peritrophin 1**	**peritrophin**	***A. gambiae***
532	7	6	10	23	agCP1729	structural constituent of ribosome	*A. gambiae*
165	10	7	7	24	agCP7766	structural constituent of ribosome	*A. gambiae*
199	9	5	10	24	agCP4384	structural constituent of ribosome	*A. gambiae*
17	12	3	10	25	agCP8340	structural constituent of ribosome	*A. gambiae*
528	6	7	13	26	agCP4228	structural constituent of ribosome	*A. gambiae*
**99**	**16**	**3**	**8**	**27**	**agCP3123**	**serine-type endopeptidase**	***A. gambiae***
**230**	**1**	**7**	**19**	**27**	**agCP2518**	**vitellogenin**	***A. gambiae***
139	6	5	17	28	ebiP415	structural constituent of ribosome	*An. gambiae*
229	8	9	11	28	agCP8207	structural constituent of ribosome	*An. gambiae*
**18**	**25**	**2**	**4**	**31**	**agCP11264**	**serine-type endopeptidase**	***A. gambiae***
**15**	**19**	**5**	**10**	**34**	**Ef1alpha48D**	**elongation factor**	***D. melanogaster***
269	10	6	18	34	agCP9994	structural constituent of ribosome	*A. gambiae*
4	17	5	13	35	agCP9893	structural constituent of ribosome	*A. gambiae*
521	7	14	15	36	agCP8317	structural constituent of ribosome	*A. gambiae*
91	13	12	26	51	agCP11873	structural constituent of ribosome	*A. gambiae*
228	19	16	30	65	agCP9509	structural constituent of ribosome	*A. gambiae*
**3**	**31**	**21**	**37**	**89**	**agCP10095**	**unknown**	***A. gambiae***
**1**	**27**	**19**	**44**	**90**	**LP07070**	**unknown**	***D. melanogaster***

25 of the 149 contigs containing more than five ESTs represent cDNAs differentially expressed among the three libraries (Table 6). We detected three main patterns of expression among these genes. These include 1) up-regulation in the IRB library relative to any other library, 2) up-regulation in both of the RB and IRB libraries relative to S, and 3) down-regulation in the RB and IRB libraries and/or up-regulation in the S library. Gene products up-regulated at 30 h PI with *P. berghei *included AS 996, agCP14019, an apparent cathepsin B; AS 230, Vitellogenin but not AS 447, the other vitellogenin gene 3' end contig (see below); AS 24, a probable vitelline membrane protein; AS 139, ribosomal protein L44; AS 475, mucin; AS 145, a high molecular weight (HMW) kininogen; AS 313, cytochrome c oxidase; and AS 270, which is similar to GenBank accession #BM600177, an unknown gene product sequenced in the Celera Genomics *A. gambiae *EST project [1].

**Table 6:** ASSEMBLED SEQUENCES WITH STATISTICALLY SIGNIFICANT DIGITAL NORTHERN VALUES

**AS**	**Sequence similarity; Description**	**S**	**RB**	**IRB**	**S (norm)**	**RB (norm)**	**IRB (norm)**	**AC S-RB**	**AC S-IRB**	**AC RB-IRB**	**R**
996	agCP14019; similar to *Aedes aegypti *vitellogenic cathepsin-B like protease (VCB).	0	0	5	0	0	*29*		**0.015**	**0.03**	***0.007***
24	no known predicted protein; shares 96% nucleotide identity with transcript ENSANG00000021567, a gene product belonging to the vitelline membrane protein family	0	1	12	0	*9*	*72*	0.240	***0***	***0.004***	***0***
139	ebiP415; Ribosomal protein L44	6	5	17	*35*	*44*	*102*	0.132	***0.005***	**0.017**	**0.034**
475	agCP12050; AgMuc1	2	3	12	*12*	*26*	*72*	0.138	***0.002***	**0.024**	***0.012***
145	agCP6338; protein of unknown function containing a HMW kininogen protein domain	1	2	8	*6*	*18*	*48*	0.172	***0.008***	**0.046**	**0.034**
313	CG14235; Cytochrome c oxidase	1	2	8	*6*	*18*	*48*	0.172	***0.008***	**0.046**	**0.034**
270	no known predicted protein; shares sequence identitiy with transcript 17000687051196 A.Gam.ad.cDNA.blood 1 of unknown function	2	3	10	*12*	*26*	*60*	0.138	***0.007***	**0.042**	**0.042**
230	agCP2518; Vitellogenin 1	1	7	19	*6*	*61*	*114*	***0.005***	***0***	**0.024**	***0***
98	agCP4445; hydrogen-transporting two-sector ATPase	0	3	4	0	*26*	*23*	**0.038**	**0.030**	0.119	**0.035**
521	agCP8317; Ribosomal protein L13	7	14	15	*41*	*122*	*90*	***0.005***	**0.018**	**0.043**	**0.039**
447	agCP2518; Vitellogenin 1	0	10	8	0	*87*	*48*	***0***	***0.002***	0.034	***0***
35	Glutathione S-transferase D1-6	0	4	3	0	*35*	*17*	**0.015**	0.060	0.081	**0.024**
537	agCP5224; Glycine hydroxymethyltransferase	0	5	2	0	*44*	*11*	***0.006***	0.123	**0.034**	**0.009**
273	agCP12503; ATP synthase B chain mitochondrial precursor (FO-ATP synthase subunit B)	1	6	3	*6*	*52*	*17*	***0.010***	0.123	**0.032**	**0.038**
516	agCP3409; AgPer1	8	10	3	*46*	*87*	*17*	**0.046**	**0.045**	***0.003***	**0.029**
8	agCP8133; Ribosomal protein L38e	14	6	3	*81*	*52*	*17*	0.076	***0.003***	**0.032**	**0.025**
208	agCP7801; ATP dependent RNA helicase	5	1	0	*28*	*9*	0	0.113	**0.017**	**0.166**	**0.030**
28	agCP11956; Serine protease G13	17	3	0	*98*	*26*	0	**0.008**	***0***	**0.027**	***0***
591	agCP1095; peptide with no known function, is part of the protein Family mucin 4 tracheobronchial mucin fragment, shares weak identity to *Cryptosporidium parvum *mucin-like glycoprotein 900	6	1	0	*35*	*9*	0	0.079	***0.009***	0.17	***0.013***
113	agCP10139; 65 aa peptide of unknown function containing no known protein domains except signal peptide and transmembrane regions.	8	0	0	*46*	0	0	***0.010***	***0.002***		***0***
18	agCP11264; Trypsin 1	25	2	4	*145*	*18*	*23*	***0***	***0***	0.125	***0***
408	17000687367332 A.Gam.ad.cDNA1; EST of unknown function	5	0	0	*28*	0	0	**0.047**	**0.017**		***0.008***
13	gene ENSANGG0000002077 6; gene located 4 kb 3' to the Peritrophin 1 that encodes a protein with a chitin binding domain	16	2	5	*93*	*18*	*29*	***0.004***	***0.006***	0.104	***0.007***
99	agCP3123; Chymotrypsin 2	16	3	8	*93*	*26*	*48*	***0.01***	**0.025**	0.069	**0.054**
170	agCP12309; putative homolog of *Drosophila melanogaster *Heat shock 70 kDa protein cognate 4 (Heat shock 70 kDa protein 88E)	7	0	4	*41*	0	*23*	**0.017**	0.086	**0.050**	**0.028**

Actual EST counts (S, RB and IRB) and the normalized number of ESTs (S (norm), RB (norm), and IRB (norm)) are given for each cDNA library. Assembled sequences were identified as differentially expressed among the three libraries if the R statistic was less than 0.05 (Stekel *et al*. 2000). The Audic and Claverie statistic (AC < 0.05) was used to identify libraries in which the assembled sequence was statistically significantly differentially expressed (Audic and Claverie, 1997). Pairwise comparisons between libraries are indicated by AC S-RB, AC S-IRB, and AC RB-IRB, respectively. All statistics were calculated with the IDEG6 Tool (Romualdi *et al*., 2003). Cells with characters in bold (underlined), bold (italics) and bold indicate statistical values equal to zero, less than 0.01, and less than 0.05, respectively. Cells with characters in *italics* (only), represent different levels of gene expression ranging from no detectable transcripts (*smallest*) to 145 transcripts (*largest*) detected in a library.

**Figure 3:**
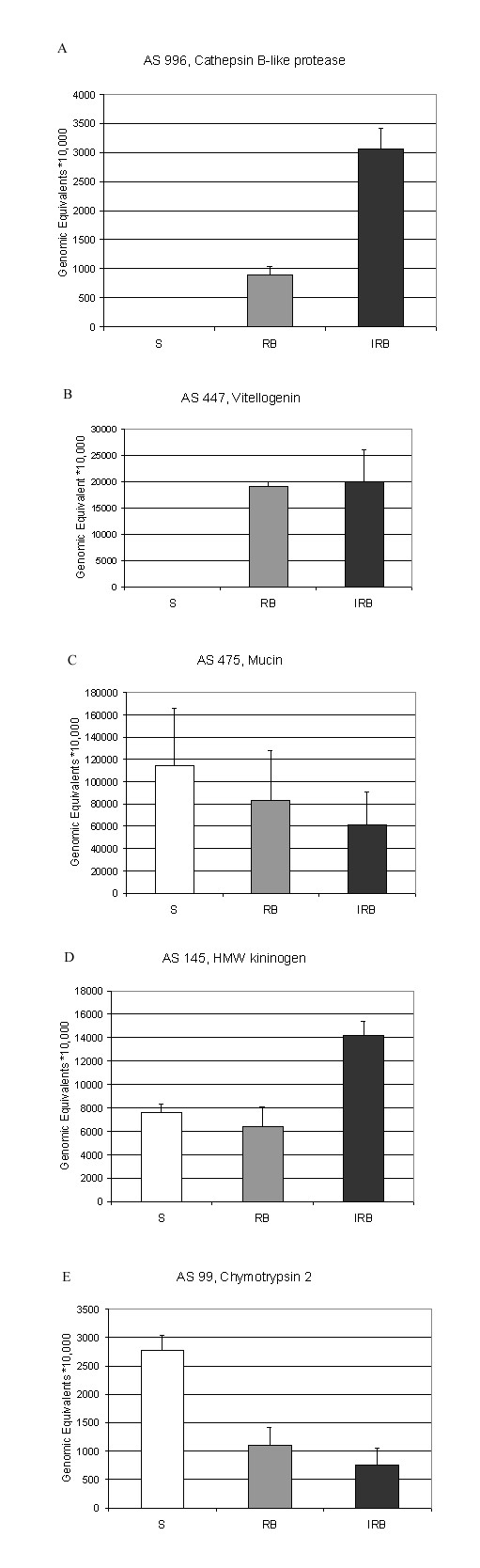
qRT-PCR of selected genes identified as differentially expressed in *A. gambiae *abdomens by digital Northern analysis. A. AS 996, a cathepsin B-like protease; B. AS 447, Vitellogenin; C. AS 475, Mucin; D. AS 145, a putative high molecular weight kininogen; and E. AS 99, Chymotrypsin 2. Transcript abundances were normalized against the abundance of RP S7 under the same condition and shown as genomic equivalents * 10,000 for S, RB, and *P. berghei*-infected (IRB) adult female mosquitoes 30 h post treatment. The arithmetic means of three biological replicates SEM are shown. ANOVA was used to compare the means, and a *post hoc *Tukey test was used to make pairwise comparisons. Statistically significant differences, P < 0.05 are discussed in the text.

AS 996, a cathepsin B, sharing marginally-significant sequence identity with the *Ae. aegypti *vitellogenic cathepsin B-like protease, VCB, [75], also appeared to be upregulated in the IRB library. In *Ae. aegypti*, VCB is secreted maximally from the fat body at 24 h PBM and accumulated by developing oocytes. During embryogenesis VCB degrades vitellin, the stored form of vitellogenin. qRT-PCR of AS 996 showed that it is induced following a blood meal, and increased further in infected blood-fed mosquitoes in comparison with naïve blood-fed mosquitoes (Figure 3A). Ribeiro [15] also found using a digital Northern approach that this gene is up-regulated in whole adult female mosquitoes 24 h PBM. It is possible that the observed increase in this cathepsin B may be responsible for part of the decrease in vitellogenin protein observed late in the first gonotrophic cycle and during the second gonotrophic cycle following infection with *Plasmodium *[18]. It is also possible that this cathepsin B may have an immunity function.

Female mosquitoes synthesize large quantities of vitellogenin in the first day following a blood meal. Consequently, it was expected that the RB and IRB libraries would show increases in vitellogenin (Vg) gene transcript abundance above the S library. Both AS 230 and AS 447 share sequence identity with Vg but represent non-overlapping sequences from the 5' and 3' ends of the genes, respectively. When these two contigs were analyzed together, Vg appeared to be expressed at significantly higher abundance in the RB library than in S, as expected, and not to be repressed and/or degraded within 30 h of *P. berghei *infection (Table 6). This result is supported by qRT-PCR analysis (Figure 3B). Since Ahmed *et al*. [18] showed that *P. yoelii nigeriensis *infection of *A. gambiae *results in fecundity reduction, due, in part, to reduction in vitellogenin mRNA accumulation, we might have expected to see a slight, though statistically insignificant reduction in Vg mRNA abundance in the IRB library by 30 hr PBM, even at the lower temperature required for development of *P. berghei *(19°C vs. the 24°C used by Ahmed et al.) ^2 ^analysis showed that AS 230 is expressed in significantly higher abundance than AS 447 in both the RB and IRB libraries, 0.05 > P > 0.025 and P < 0.001, respectively. However a goodness of fit test showed that the under-representation of Vg mRNA 3' ends is exaggerated significantly in the IRB library, G = 26.3, P < 0.01 [76]. We can not currently explain this observation, except to suggest that Vg transcripts may be degraded from their 3' ends in response to *Plasmodium *infection.

AS 24 also appears to be up-regulated in response to *Plasmodium *infection. BLASTN analysis showed that its nucleotide sequence is 96% identical to the *A. gambiae *transcript ENSANG00000021567, a gene product belonging to the vitelline membrane protein family. Insect orthologs of AS 24 include both the *Drosophila *Vm34Ca protein and the *Ae. aegypti *vitelline membrane protein 15A-1. In *Ae. aegypti*, 15A-1 mRNA is most abundant between 30 and 45 h PBM [77]. Our qRT-PCR analysis showed that this gene is induced following a blood meal (data not shown).

*P. berghei *invasion of *An. stephensi *midguts results in damage to invaded epithelial cells and their extrusion into the midgut lumen [41,78]. This may induce an inflammatory response. AS 475 is 100% identical at the amino acid level to the previously identified midgut-specific, membrane-bound mucin AgMuc1. Associated with the apical microvilli on the midgut, this mucin contains a putative GPI-anchor and two hydrophobic domains. This result suggested that there may be a link between this membrane mucin and signal transduction following damage to the epithelium [79]. Membrane mucins also act as physical barriers protecting the free surface of the cell [80]. Therefore, increased mucin gene expression may serve as a protective response to parasitic invasion. Ribeiro [15] found that this mucin is up-regulated in blood-fed mosquitoes 24 h PBM [15]. Our qRT-PCR analysis showed no difference in gene expression PBM or PI (Figure 3C).

AS 145, putatively identified as agCP6338, contains a HMW kininogen protein domain. This domain is a signature for a family of similar inflammatory response proteins in vertebrates. HMW kininogen is a component of the vertebrate kinin system, a pathway involved in inflammation and pain responses to cell damage [44]. Both digital Northern analysis and qRT-PCR (Figure 4D) identified AS 145 as being induced only after *Plasmodium *infection. Damage to the midgut epithelium caused by invasion and penetration of *P. berghei *ookinetes may have been responsible for inducing AS145.

In contrast to other gene products induced in mosquito abdomens by infection with *P. berghei*, AS 170 appears to be repressed following a blood meal to levels below those found in sugar-fed females though it is well represented in the IRB library. This assembled sequence, which is derived completely from 5' UTR, not from protein-coding sequence, is highly identical to GenBank Accession # BM653334, identified among their *A. gambiae *ESTs by Celera Genomics [1]. BM653334 was assembled into TC10892 in the TIGR gene indices, a tentative consensus sharing identity with the *Drosophila *70-kDa heat shock protein, Hsp70. Hsp70s are molecular chaperones highly conserved in all organisms. While Hsp70s are induced in response to heat shock and other stresses, they also function in many normal cellular processes including protein translation, translocation, folding and quality control, as well as repression of cell growth and apoptosis through specific protein-protein interactions [81]. Clearly, induction of this gene post IRB could be a stress response to *Plasmodium *infection. The interactions of mosquito Hsp70s with both mosquito and parasite proteins deserve further study.

Genes up-regulated following a blood meal could be divided into two groups, genes whose transcription is unaffected by *P. berghei *infection at 30 h PI, and genes whose transcription is apparently repressed. Gene products grouped into the first category include AS 98, hydrogen-transporting two-sector ATPase; AS 521, ribosomal protein L13; and AS 447, the second Vitellogenin 3' end contig. In contrast, gene products in the latter category include AS 35, glutathione S-transferase 1–6; AS 537, glycine hydroxymethyltransferase; AS 273, ATP synthase b; and AS 516, Peritrophin 1.

AS 516 is 98% identical at the amino acid level to Peritrophin 1, a midgut peritrophic matrix (PM) protein, and maps *in silico *to the same location on chromosome 2L as Peritrophin 1. Peritrophin 1 mRNA is present in sugar-fed females at 5 days post-eclosion, but is induced 12–24 h PBM [82]. Our digital Northern analysis showed that AS 516 is induced by at 30 h PBM, but repressed in response to *Plasmodium *infection. Ookinetes may partially inhibit PM formation, facilitating their penetration through it to gain access to the midgut epithelium. This is unsurprising, given that ookinetes produce locally-acting chitinases that are required for PM penetration and midgut invasion [83-85], and that they are targets for transmission blocking vaccines [86].

Almost half of the differentially expressed genes are down-regulated PBM. AS 113 and AS 408, two unknown gene products; AS 13, a peritrophin-like protein; AS 18, Trypsin 1; and AS 99, Chymotrypsin 2, are significantly less abundantly expressed in the mosquito abdomen at 30 h PBM than in S females and not affected further by ookinete invasion. qRT-PCR (Figure 3E, see below) verifies this expression pattern for Chymotrypsin 2. Genes repressed PBM may also include a AS 591, a gene product with no known function but which is part of the mucin 4, tracheobronchial mucin fragment protein family, and AS 208, an ATP dependent RNA helicase, although the differences in abundance of these transcripts between S and RB are not statistically significant.

AS13 shares greatest amino acid identity with Peritrophin 1. However, it shares greater nucleotide identity with ENSANGG00000020776, a gene located 4 kb downstream of Peritrophin 1, and is likely to have been derived from the latter. AS 18 and AS 99 share identity with two enzymes involved in digestion of the blood meal. AS 18 is 100% identical at the amino acid level to Trypsin 1. Northern blots showed that transcripts of Trypsin 1, P35035, are present in adult female mosquitoes by 4 h PBM, increase rapidly until 12 h, peak at 16 h, remain at this plateau until 24 h, then decrease steadily until 40 h, and drop to baseline levels by 48 h PBM [87,88]. Trypsin 1 is also expressed in S females until 5 days post eclosion, but not at the high levels exhibited by Trypsin 4 [87]. Therefore it is not entirely clear why we found more transcripts of Trypsin 1 than of Trypsin 4 in the S library, and more in the S than in the 30 h PBM library. AS 99, another trypsin-like SP, is 97% identical to agCP3123, previously identified as the Chymotrypsin 2 precursor, Anchym2 [89]. This gene is expressed in the midgut at 12 h PBM and remains abundant until 48 h. Anchym2 transcripts are undetectable in S females. Using qRT-PCR, we found that AS 99 transcript abundances are reduced in both RB and IRB mosquitoes relative to S females (Figure 3E), though Ribeiro [15], also using transcript frequency analysis, found that both Trypsin 1 and Chymotrypsin 2 are up-regulated in whole adult female *A. gambiae *PBM. Since trypsin acitivity is known to be age-dependent in *A. gambiae *females [90], it is possible that these serine protease genes may be transcribed at higher levels in younger adult females than after the peak of proteolytic activity following a blood meal.

AS 28, agCP11956, SP G13, appears to be down-regulated PBM and further repressed following ookinete invasion. However it was shown not to be blood meal responsive in a previous study [34].

Housekeeping gene products involved in normal cellular maintenance may also be differentially expressed following septic injury. AS 28 and AS 170, the hsp70 homolog discussed above, are potential examples of two such proteins. Two assembled sequences corresponding to housekeeping genes up-regulated following *P. berghei *infection, AS 313 and AS 139, share sequence identity with a cytochrome c oxidase subunit and ribosomal protein L44, respectively. In contrast, the only housekeeping gene product down-regulated following *P. berghei *infection is AS 8 which shares amino acid identity with ribosomal protein L38e. The only gene identified as significantly more abundantly expressed in the mosquito abdomen PBM but unaffected by the presence of *Plasmodium *infection was AS 98, a hydrogen-transporting two-sector ATPase. Increased abundances of oxidative phosphorylation proteins may be linked to apoptosis or to increased metabolic demands placed on invertebrate hosts by invaders. The roles of these housekeeping gene products in immunity remain to be established.

Several *A. gambiae *genes identified in this study as being differentially regulated (Table 6) have potentially also been studied in microarray experiments performed in *A. stephensi *under generally similar experimental conditions [17,37]. Abraham *et al*. studied *A. stephensi *genes primarily from a cDNA library enriched for genes expressed in mosquito midguts containing early oocysts of *P. berghei *[17] and identified 226 EST contigs likely to have been of mosquito origin. None of these had clear identity with any of the putative differentially expressed *A. gambiae *genes in Table 6. Xu *et al*. studied *A. stephensi *genes differentially regulated between 6 h and 20 d PI with *P. berghei *[37]. Though the strategy used by Xu *et al*. in making the subtraction cDNA libraries assayed in the microarray was expected to have biased their identified mosquito genes towards ones upregulated PI and did bias mosquito genes towards ones upregulated between 20 h and 20 d PI, a chymotrypsin 2 precursor was identified as being expressed more abundantly at earlier times than at later ones. Since their study did not include a 0 h PI time point, we cannot be certain that there is an actual discrepancy between our data and theirs. They found a peak in NOS activity at 40 h PI though we have no NOS ESTs with which to compare their data. Since Xu *et al*. did not test RNA from uninfected mosquitoes and tested only one time point, 20 h, at all close to our 30 h we can not thoroughly evaluate the single identified gene overlap between the two data sets. In addition to the facts that experimental conditions varied among all three data sets and that at lease one of these data sets was small, it is possible that *A. gambiae *and *A. stephensi *have only partially overlapping repertoires of transcriptionally regulated responses to infection with *P. berghei*, simply because they are not extremely closely related anophelines. Though they are both classified within the subgenus *Cellia*, they are grouped differently within the subgenus, *A. gambiae *in the *Pyretophorus *series and *A. stephensi *among the *Neocellia*.

## Conclusion

We have described 1975 genes expressed in the abdomens of adult female *A. gambiae *mosquitoes, 13% of which are not predicted by the genome sequence or by identity with known genes in other organisms. The latter provide important information for further *A. gambiae *genome annotation. All together these genes form a resource likely to be very useful for annotation of genes in other organisms. While *D. melanogaster *provides a model for many kinds of biological and bioinformatic analyses, its genome has been highly streamlined, more so than that of *A. gambiae*. Consequently, the *A. gambiae *genome and its associated genomic resources including its EST collections may sometimes prove more useful than *Drosophila *for annotation of genes in taxonomically distant organisms.

We have identified several genes as being induced following blood-feeding and/or *P. berghei *infection using the digital Northern technique. Limitations on this approach include the numbers of ESTs obtained from each library, the limited number of time points or physiological states that can be examined, and the requirement that their mRNAs be of intermediate or high abundance in at least one of the conditions studied. Thus some, indeed, many blood-feeding and immune-responsive genes may not have been identified, simply because they are expressed at low levels despite the importance of their roles in these processes. In addition, transcript abundances do not always correlate highly with protein levels. Some mRNAs have high turnover rates while others may be stabilized yet not translated except under specific conditions. Multiple, as well as more targeted approaches may be required before all gene products involved in responses to infection with *Plasmodium *are identified. Nevertheless, the ESTs obtained from the three different cDNA libraries have provided at least a rudimentary catalog of genes expressed in the abdomens of adult female *A. gambiae *harvested 30 h after they had fed on sugar, on blood or on blood infected with *P. berghei*. We should acknowledge some caution in the interpretation of cases where gene expression patterns in this study differ from those of comparison studies. This study was based on whole mosquito abdomens, which contain a multitude of organs, tissues and cell types and some comparison studies may involve either whole mosquitoes or subsets of the organs and tissues found in abdomens. Despite the limitations of the digital Northern portion of our study, we have identified several gene products as candidates for involvement in *Plasmodium*-immunity processes. These should be studied further. In addition, the sequences in the three cDNA libraries will certainly inform more detailed microarray and qRT-PCR studies of *A. gambiae *gene expression in both physiologically normal and *Plasmodium*-infected females.

## Methods

### *Mosquitoes and *P. berghei *infections*

*Plasmodium *infection susceptible (4arr) and transforming ookinete-encapsulating (L-35) strains of *A. gambiae *were reared under standard conditions 25°C, 70% humidity, 12 h light/dark. Adults were maintained on 20% sucrose until they were five to seven days post-eclosion. They were then blood fed on naïve and *P. berghei*-infected rats and maintained under similar conditions except that the temperature was lowered to 19–20°C to be permissive for development of *P. berghei *[91].

Female white rats (*Rattus norvegicus*) were maintained in the Freimann Life Sciences Center according to protocols established by the University of Notre Dame IUCAC. They were infected by intraperitoneal injection with 4 × 10^7 ^*P. berghei *gametocytes of the ANKA 2.34 clone (gift from Marcelo Jacobs-Lorena) suspended in 10% DMSO. Rat parasitemia levels were determined from Giemsa-stained thin blood smears prepared from eye blood. Unfixed fresh eye blood samples were examined for gametocyte exflagellation, and exflagellation levels calculated as the number of events per 20 random microscopic fields at 400X magnification.

Female mosquitoes were blood fed on Plasmodium-infected anaesthetized rats. To determine infection intensity and the time by which the majority of *P. berghei *ookinetes had penetrated to the basal lamina of the mosquito midgut epithelium and were transforming to oocysts, midguts of infected L-35 strain females were dissected in 1% formaldehyde in PBS at 30, 36 and 42 hours PBM, then transected longitudinally to remove the food bolus, washed in PBS and mounted flat on microscope slides [92,93]. Slides were examined microscopically and encapsulated parasites counted using bright field illumination and 400 × magnification. A two-way ANOVA was used to compare the mean numbers of parasites per midgut at the three times and a post hoc Tukey test was employed to detect differences in the means [76,94].

### cDNA library construction

5–7 day old female mosquitoes of the 4arr strain were 1) sugar fed (S library), 2) blood fed (RB library), and 3) *P. berghei*-infected (IRB library). Mosquitoes were immediately transferred to 20°C and incubated for 30 hours. Parasitemia of the rat used for these infections was 11.1%. Mean mosquito infection prevalence was 83%. The infection intensity ranged from 2 to 23 with an average of 9 parasites per midgut.

Blood and sugar-fed females were flash frozen in liquid nitrogen at 30 h PBM, or the equivalent age in the case of the S females, then vortexed at -20°C to sever abdomens from heads and thoraces, similarly to [86]. Abdomens were collected at -20°C and total RNA extracted using TRIzol (MRC, Inc.) according to the manufacturer's instructions. Poly-A^+ ^mRNA was isolated using the PolyA Tract mRNA Isolation System (Promega). S, RB and IRB cDNA libraries were constructed using the SMART™ cDNA Library Construction Kit (Clontech) from 1.54, 3.82 and 2.1μg of poly-A^+ ^mRNA, respectively. Unless otherwise stated, all reagents used were those provided in the kit. Reverse transcription of mRNA was for 1 hr at 42°C using Superscript II Reverse Transcriptase (Invitrogen) and a modified oligo-dT primer, CDSIII/3' PCR Primer:5' -ATTCTAGAGGCCGAGGCGGCCGACATG-d(T)30-3'(Invitrogen), to prime the first strand synthesis reaction. This primer contains the Sfi IB restriction site used for directional cloning. An additional oligonucleotide, either the SMARTIII™ Oligonucleotide, 5'-AAGCAGTGGTATCAACGCAGAGTGGCCATTATGGCCGGG-3'(Slibrary), or the SMART IV TM Oligonucleotide, 5'-AAGCAGTGGTATCAACGCAGAGTGGCCATTACGGCCGGG-3'(RB and IRB libraries), contains the Sfi IA restriction site followed by 3 guanines. Second strand synthesis of cDNAs was conducted in a 100 l volume with 11.0 l first strand cDNA, 0.2 M of the indicated 5' and 3' oligonucleotides, 1X Advantage 2 PCR Buffer, 1X dNTP Mix, and 1X Advantage 2 Polymerase Mix. These reactions were performed on a Perkin-Elmer 9600 Thermocycler using the following cycling conditions: 72°C for 10 min, 95°C for 20 s, followed by 3 cycles of 95°C for 5 s and 68°C for 8 min. Primer extended cDNAs were visualized on an ethidium bromide stained 1.25% agarose, 1X TBE gel for quality assessment. They appeared as homogeneous smears ranging from 100 bp to 5 kb. mRNA aliquots not used for cDNA synthesis and cloning were also subjected to PCR amplification using Taq Polymerase and visualized on the same gel. The absence of any visible product on the gel confirmed that genomic DNA did not contaminate these mRNA populations.

Following proteinase K digestion and phenol:chloroform extraction, the amplified cDNAs were digested with 10 μl Sfi I (20 U/μl) at 50°C for 2 h and size fractionated using CHROMA SPIN-400 columns (Clontech). The first three to four fractions containing cDNAs longer than 500 bp were pooled, ethanol precipitated, and concentrated in 4.0 l nuclease free water (Gibco, UltraPure). These cDNAs were directionally cloned into Sfi I digested TripIEx2 (Clontech), and packaged using Gigapack III Gold Packaging Extract (Stratagene) according to the protocol provided. Packaged recombinant phages were incubated with log phase *E. coli *XL1-Blue cells (Stratagene), plated and library titers determined. All three libraries were plated at 100 and 1000 pfu/plate.

White plaques were isolated and recombinant phages eluted overnight in 100 l SM buffer (0.1 M NaCl, 0.01 M MgSO_4_.7H_2_O, 0.05 M Tris-HCl (pH 7.5), 0.01% (w/v) gelatin). The inserts were amplified via PCR using 5' and 3' vector specific primers; 5' LD Amplimer Primer, 5'-CTCGGGAAGCGCGCCATTGTGTTGG-3' and 3' LD Amplimer Primer, 5'-ATACGACTCACTATAGGGCGAATTGGC-3' (Invitrogen). Amplification reactions contained 0.4 l eluted phage, 0.03 pmol of each primer, 1X Taq Polymerase Buffer (Invitrogen), 3 mM MgCl_2_, 1 mM of each dNTP, and 0.2 U Taq Polymerase (Invitrogen) in a total volume of 25 ml. Reactions were performed in 96-well plates on a Perkin-Elmer 9700 Thermocycler using the following cycling conditions; initial denaturation at 95°C for 5 min, followed by 25 cycles of denaturation at 94°C for 30 s and annealing/elongation at 70°C for 2 min, and a final elongation step at 68°C for 3 min. Seven samples were chosen randomly from each 96-well plate amplified, and 5 ml of the reaction was electrophoresed on an ethidium bromide stained 1% agarose, 1X TBE gel to confirm that the PCR was not contaminated and that no primer dimers could be visualized (data not shown). 704 PCR-amplified cDNA clone inserts were visualized on ethidium bromide stained 1% agarose, 1X TBE gels, and their insert sizes determined using the KODAK Digital Science 1D software (Scientific Imaging Systems; data not shown).

### cDNA clone sequencing and EST assembly

cDNA clones were picked at random from the S, RB and IRB abdomen libraries and their 5' end sequences obtained through single-pass sequencing of the PCR-amplified inserts using the ABI PRISM Big Dye Terminators 3.0 Cycle Sequencing kit (ABI). All sequencing reactions were performed in 384-well plates on a Perkin-Elmer 9700 Thermocycler using the following cycling conditions; initial denaturation at 94°C for 4 min, followed by 25 cycles of denaturation at 94°C for 10 s, annealing at 50°C for 5 s, and elongation at 60°C for 4 min. Each reaction contained 0.7 ml PCR product, 7.4 pmol of the 5' LD Amplimer Primer, 1× Sequencing Buffer (400 mM Tris pH 9.0, 10 mM MgCl_2_), and Big Dye (ABI) in a total volume of 7 ml. Reaction products were ethanol precipitated, resuspended in 20.0 ml HiDi formamide (ABI) and electrophoresed on an ABI 3700 Sequencer.

Sequences were trimmed of low quality and vector sequence, then screened to remove mitochondrial sequences using the SeqMan II software (DNASTAR, Inc.), prior to contig assembly. The options employed for SeqMan II assembly were match size = 12 bases, minimum match % = 80, minimum sequence length = 100, maximum added gaps per kb in contig = 70, maximum added gaps per kb in sequence = 70, maximum register shift difference (maximum base pair separation) between matches = 70, gap penalty = 0, and gap length penalty = 0.7. Consensus sequences derived from the alignment of multiple ESTs were defined as contigs, whereas ESTs that did not assemble into a cluster were defined as singletons. Consensus sequences were called by trace evidence, the majority percentage = 75, using the quality weights option.

### Bioinformatic analysis

Bioinformatic analysis was initiated by subjecting consensus contig and singleton sequences to several blast searches. Initially, sequences were tested against the *A. gambiae *genome using BLASTN 2.2.4 [95,96]. The significance cutoff was chosen as E < 1 × 10^-4^. Sequences were then tested against the non-redundant nucleotide database in GenBank using BLASTX 2.2.4, at the same URL. Sequences that failed to yield significant BLASTX matches were retested against the same database using BLASTN. Finally, sequences lacking any significant BLAST hits were tested against dbEST using BLASTN.

Gene product identities were inferred from BLAST hits and the annotations provided for *A. gambiae *and *D. melanogaster *clones in public databases including The Institute for Genomic Research (TIGR) Gene Indices [97] and GadFly Genome Annotation Database in FlyBase [98-100]. Putative molecular functions of the gene products were determined using KEGG [101] and assigned to categories established by the Gene Ontology Consortium, GOC [102,103]. Gene products were also assigned to hybrid biological process categories by combining the categories used by the GOC and by [1].

### Digital northerns

Each contig represents an expressed gene and the number of sequences within a contig represents its transcript abundance. As in [104], only contigs containing more than five ESTs were used for transcript profiling. Gene expression profiles were created by tabulating the frequencies of cDNAs corresponding to a particular gene in each library and then compared among the three experimental groups. Genes were identified as differentially expressed using the R Statistic. Differences between libraries were determined using the Audic and Claverie pairwise comparison statistic calculated using IDEG6 [104].

### qRT-PCR

Quantitative real-time PCR (qRT-PCR) analysis was performed using SYBR Green I (Applied Biosystems) technology in order to validate data obtained from the digital Northerns,. The Primer Express v. 1.5 software (Applied Biosystems) was used to design primer sets for the following 8 transcripts: AS 24 (Forward 5'-GAAGTAGCGAGAGACAGCATCGA-3', Reverse 5'-TACGCTTCGGAGGTCAGTTACTG-3'); AS 99 (Forward 5'-TTGCTGTCTCGGTACTCCTAG-3', Reverse 5'-GGTTGACGTAGTTGTCGTCCA-3'); AS 113 (Forward 5'-TGTTAGTCGCCCTGATGCTG-3', Reverse 5'-TCAATGTTATGGGTACACCTTGTGT-3'); AS 145 (Forward 5'-TGGCGATCTTTGTCATCGTG-3', Reverse 5'-GATGACCGTGTTGACCACCAT-3'); AS 447 (Forward 5'-TCCACTGCCGTGACGCT-3', Reverse 5'-TCCCTTGCGGATCTGCTG-3'); AS 475 (Forward 5'-TGCCCCACAGGATGTGAAA-3', Reverse 5'-ATCGACATTGCCACGTATGC-3'); AS 996 (Forward 5'-GTCGGGCGATTCCAATGA-3', Reverse 5'-TGTAACCGGGCTGGCAAA-3'); and the ribosomal protein S7 gene, RP S7, (Forward 5'-CATTCTGCCCAAACCGATG-3', Reverse 5'-AACGCGGTCTCTTCTGCTTG-3'). RP S7 [106] is the internal control currently most widely used in studies of *A. gambiae *gene expression [34,35,46,105]. All amplifications and fluorescence quantifications were performed using an ABI 7700 Sequence Detection System and associated Sequence Detector Software v. 1.7 (Applied Biosystems). Standard curves were generated using 10-fold serial dilutions of *A. gambiae *strain 4Arr genomic DNA extracted using the Qiagen DNeasy Tissue Kit protocol for animal tissues (Qiagen, Inc.). The DNA dilutions ranged from 115 to 0.0115 ng per reaction. Total RNA was extracted from rat blood-fed and *P. berghei*-infected whole adult female mosquitoes using TRIzol (MRC, Inc.) RNA samples were incubated with 1.0 μl of DNase I in the supplied DNase I buffer (Invitrogen) for 15 min at room temperature to remove contaminating DNA. The DNase I was then inactivated by addition of 4.0 μl of 25 mM EDTA, pH 8.0 and the total RNA reisolated from TRIzol. RNA sample quality was evaluated by electrophoresis on 1% agarose, 1 × TAE gels, and ethidium bromide staining of the gels. 5 g samples of RNA were reverse transcribed at 42°C for 1.5 h using Superscript II Reverse Transcriptase and oligo(dT) primer (both from Invitrogen) to prime first strand synthesis. qRT-PCR reactions were performed in duplicate in total volumes of 25 l containing 12.5 l of SYBR Green I PCR Master Mix, 300 nmol of each gene-specific primer, 50 ng of first strand cDNA template, and nuclease free water (Gibco, UltraPURE). All qRT-PCR reactions were performed using the following conditions: 50°C for 2 min, then 95°C for 10 min followed by 45 cycles of denaturation at 95°C for 15 s, annealing and extension at 60°C for 1 min. Amplification plots were generated as fluorescence of SYBR Green I (Rn) vs. PCR cycle number using the Sequence Detector Software v. 1.7 (Applied Biosystems). The abundance of each gene product in an RNA sample was estimated from its standard curve and normalized against the RP S7 transcript abundance in the same RNA sample. Expression levels were represented as genomic equivalents × 10,000. All comparisons were replicated on at least three biological samples, and the means and SEMs reported.

## Abbreviations

AS, assembled sequence; bp, base pairs; EST, expressed sequence tag; GST, glutathione-S-transferase; h, hour; IRB, [*Plasmodium*-] infected red blood; kb, kilobases; min, minutes; nt, nucleotides; PBM, post blood meal; PI, post-infection; RB, rat blood; S, sugar, 20% sucrose; s, seconds; SEM, standard error of the mean; SP, serine protease; Vg, vitellogenin.

## Authors' contributions

AND carried out the study with contributions from PAR, MEH, NFL and MKK. PAR and AND drafted the manuscript with contributions from NFL and FHC. All authors read and approved the final manuscript.

## Supplementary Material

Additional File 1 Gene identification and ontology of EST contigs identified in this study. Click here for file
